# Deciphering the Molecular Mechanisms behind Drug Resistance in Ovarian Cancer to Unlock Efficient Treatment Options

**DOI:** 10.3390/cells13090786

**Published:** 2024-05-04

**Authors:** Mariana Nunes, Carla Bartosch, Miguel Henriques Abreu, Alan Richardson, Raquel Almeida, Sara Ricardo

**Affiliations:** 1 Differentiation and Cancer Group, Institute for Research and Innovation in Health (i3S), University of Porto, 4200-135 Porto, Portugal; mnunes@i3s.up.pt (M.N.); ralmeida@i3s.up.pt (R.A.); 2 Institute of Biomedical Sciences Abel Salazar (ICBAS), University of Porto, 4050-313 Porto, Portugal; 3 Porto Comprehensive Cancer Center Raquel Seruca (PCCC), Portuguese Oncology Institute of Porto (IPO-Porto), 4200-072 Porto, Portugal; carla.bartosch@ipoporto.min-saude.pt (C.B.); antonio.m.abreu@ipoporto.min-saude.pt (M.H.A.); 4 Department of Pathology, Portuguese Oncology Institute of Porto (IPO-Porto), 4200-072 Porto, Portugal; 5 Cancer Biology & Epigenetics Group, Research Center of Portuguese Oncology Institute of Porto (CI-IPO-Porto), Health Research Network (RISE@CI-IPO-Porto), Portuguese Oncology Institute of Porto (IPO-Porto), 4200-072 Porto, Portugal; 6 Department of Medical Oncology, Portuguese Oncology Institute of Porto (IPO-Porto), 4200-072 Porto, Portugal; 7 The School of Pharmacy and Bioengineering, Guy Hilton Research Centre, Keele University, Thornburrow Drive, Stoke-on-Trent ST4 7QB, Staffordshire, UK; a.richardson1@keele.ac.uk; 8 Biology Department, Faculty of Sciences, University of Porto (FCUP), 4169-007 Porto, Portugal; 9 Associate Laboratory i4HB, Institute for Health and Bioeconomy, University Institute of Health Sciences—CESPU, 4585-116 Gandra, Portugal; 10 UCIBIO—Applied Molecular Biosciences Unit, Toxicologic Pathology Research Laboratory, University Institute of Health Sciences (1H-TOXRUN, IUCS-CESPU), 4585-116 Gandra, Portugal

**Keywords:** drug resistance mechanisms, ovarian cancer, platinum, taxanes, polyadenosine diphosphate ribose polymerase inhibitors, bevacizumab

## Abstract

Ovarian cancer is a highly lethal form of gynecological cancer. This disease often goes undetected until advanced stages, resulting in high morbidity and mortality rates. Unfortunately, many patients experience relapse and succumb to the disease due to the emergence of drug resistance that significantly limits the effectiveness of currently available oncological treatments. Here, we discuss the molecular mechanisms responsible for resistance to carboplatin, paclitaxel, polyadenosine diphosphate ribose polymerase inhibitors, and bevacizumab in ovarian cancer. We present a detailed analysis of the most extensively investigated resistance mechanisms, including drug inactivation, drug target alterations, enhanced drug efflux pumps, increased DNA damage repair capacity, and reduced drug absorption/accumulation. The in-depth understanding of the molecular mechanisms associated with drug resistance is crucial to unveil new biomarkers capable of predicting and monitoring the kinetics during disease progression and discovering new therapeutic targets.

## 1. Introduction

Primary debulking surgery followed by the adjuvant combination of carboplatin and paclitaxel with or without bevacizumab, which can be followed by maintenance therapies like polyadenosine diphosphate ribose polymerase inhibitors (PARPis), represents the gold standard treatment in the frontline management of advanced epithelial ovarian cancer (OC) [1,2]. The absence of macroscopic residual disease (R0) is associated with statistically significant increased overall survival (OS) and progression-free survival (PFS) [3,4]. If primary debulking surgery is not possible due to the extent of the disease or the patient’s overall health, neoadjuvant chemotherapy followed by interval debulking surgery is an alternative [5,6].

Patients with advanced OC who undergo optimal surgery and receive first-line treatment with adjuvant carboplatin plus paclitaxel can achieve a complete or partial response in the range of 50% to 81% [7,8,9] and a median PFS range of 16 to 19.3 months [10,11]. However, ultimately, about 70 to 80% of patients will experience a relapse, with the median time to recurrence being 16 months [3,12,13], and progressively develop resistance to the various therapeutic options [14]. With disease progression, complications, such as malignant ascites, bowel obstruction, and pleural effusion, occur, affecting the quality of life [15]. Thus, predicting drug resistance during treatment or delaying recurrence and improving survival after first-line treatment are significant unmet needs.

This review will focus on high-grade serous cancer (HGSC), as this is the dominant histologic subtype of OC. Despite initially being platinum-sensitive, nearly 90% of advanced-stage [Federation of Gynecology and Obstetrics (FIGO) stage III/IV] HGSC patients recur in 5 years and eventually become chemoresistant [16].

## 2. Mechanisms of Chemoresistance in Ovarian Cancer

Drug resistance in tumor cells develops through a stepwise molecular evolution, giving them a survival advantage, and can be categorized as intrinsic and acquired [17,18]. Intrinsic resistance refers to the natural ability of cancer cells to withstand and tolerate the initial exposure to treatment. Acquired resistance, conversely, refers to the capacity of neoplastic cells to adapt after drug exposure, enabling them to continue growing despite the treatment [17,18]. The amplification of cyclin 1 (CCNE1) is described as an early event (intrinsic) in the development of HGSC being mutually exclusively in breast cancer 1/2 (BRCA1/2) dysfunctional and frequently identified in patients with platinum-resistant and refractory disease [19,20]. Instead, genetic aberrations in inactivating tumor suppressor genes, such as retinoblastoma protein 1 (RB1), neurofibromatosis type 1 (NF1), RAD51 recombinase B (RAD51B), and phosphatase and tensin homolog (PTEN) are more associated with acquired resistance [21].

Intrinsic resistance mechanisms include drug degradation by metabolic enzymes [22], modifications of drug transporters and drug targets [23], poor vascularization leading to decreased intracellular drug concentration [24], extracellular matrix (ECM) interactions, and cellular metabolic processes that can promote resistance mediated by the tumor microenvironment (TME) [25]. Acquired resistance is characterized by an increase in the mutational burden and other molecular alterations that will affect drug efficacy and can be attributed to an increased drug efflux, activation of anti-apoptotic signaling pathways, and inactivation of desoxyribonucleic acid (DNA) damage repair mechanisms to evade cell death [26,27]. In addition, cancer cells can develop changes to drug targets through multiple mechanisms or bypass them through alternative pathways [28,29]. Still, it is challenging to distinguish between intrinsic and acquired mechanisms that can contribute to both categories of resistance.

One of the challenges in oncological treatment is the development of multidrug resistance (MDR), which occurs when tumor cells become resistant to structurally and mechanistically unrelated types of antineoplastic drugs, which compromises the effectiveness of therapy [30]. The MDR phenotype is characterized by a decreased drug uptake, an increased expression of drug-metabolizing enzymes, altered cell cycle checkpoint progression, changes in apoptosis or survival pathways, and deregulation of signal transduction pathways [22,23,31]. Efflux pumps, such as ATP-binding cassette (ABC) protein families, including P-glycoprotein (P-gp), multidrug resistance-associated protein 1 (MRP1), and breast cancer resistance protein (BCRP), are often overexpressed and are described as the significant contributors to MDR phenotype in many types of tumors [32]. The co-administration of anticancer drugs with MDR inhibitors can enhance therapeutic response by inhibiting drug efflux. Nevertheless, the clinical translation of drug efflux pump modulators failed due to pharmacokinetic interactions and adverse effects [30,33,34].

### 2.1. Mechanisms of Platinum Resistance in Ovarian Cancer

Platinum compounds are a type of antineoplastic agent that can prevent tumor growth by inhibiting DNA synthesis [35]. Platinum can enter cells through passive diffusion or the copper transporter (CTR1); it binds to DNA and creates intra-strand and inter-strand crosslinks that cause DNA damage, leading to cell death [36,37].

The platinum-free interval (PFI; defined as the time between the last dose of platinum-based therapy and the documented relapse) has been used for the past three decades as the primary factor to determine the likelihood of patient response if they are rechallenged with platinum [13]. OC patients are categorized into three groups based on their response to platinum-based chemotherapy. “Platinum-refractory” refers to patients who experience disease recurrence during or within four weeks after discontinuing platinum-based treatment. “Platinum-resistant” includes patients who do not respond to platinum-based therapy or experience a recurrence within six months following the conclusion of the first treatment cycle. “Platinum-sensitive” indicates patients who respond well to platinum-based chemotherapy and do not experience a recurrence within six months of finishing treatment [13,38]. However, the occurrence of platinum resistance is a continuous process, and the PFI-based cut-off to define sensitivity can be quite arbitrary as it does not consider other essential factors [13], such as whether maintenance therapy with targeted agents [39]. Therefore, in the 5th OC Consensus Conference and 2019 European Society for Medical Oncology (ESMO)–European Society of Gynecological Oncology (ESGO) guidelines, the PFI-based paradigm was revisited and partially replaced with the concept of the treatment-free interval (TFI) that subdivides patients into two categories: those eligible for platinum rechallenge and those for whom platinum is not considered an option [38,40]. A platinum rechallenge is not regarded as feasible if a progression is observed during a platinum-based treatment or early after its completion. Non-platinum compounds like topotecan, gemcitabine, anthracyclines, and paclitaxel can be alternatives. These chemotherapeutic drugs show significant activity and lower toxicity compared to combinatorial schemes [13,41,42,43,44]. TFI considers data from histopathology, BRCA1/2 mutation status, number/type of previous therapies, the outcome of prior surgery, and reported symptoms [38].

**Figure 1:**
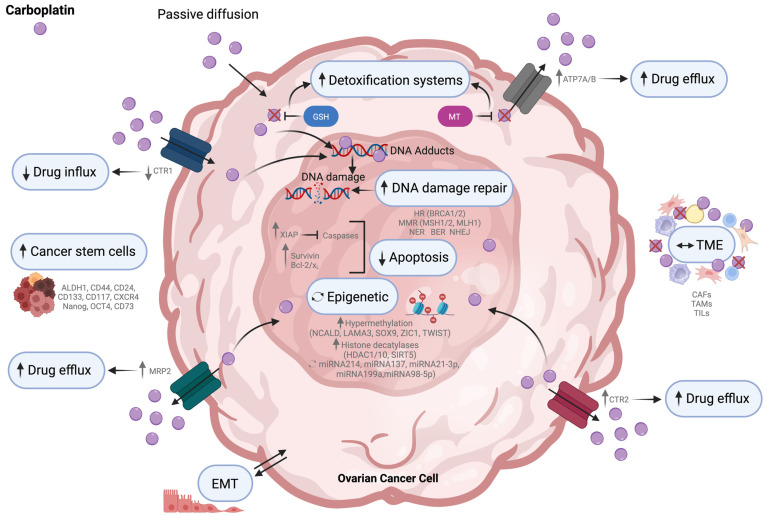
Schematic representation of the main molecular mechanisms contributing to carboplatin resistance in OC. Increased drug efflux by overexpression of MRP2/4 and ATP7A/B and decreased drug influx by CTR1/2 upregulation and detoxification systems; for example, GSH and MT can reduce the concentration of platinum inside the cells. Enhanced DNA repair mechanisms, such as alterations in BRCA1/2 and MMR genes, can overcome platinum–DNA binding. Mutations or changes in downstream signaling pathways that affect the cell’s ability to undergo apoptosis, such as increased Bcl-2/XL, XIAP, and survivin, and decreased caspases, can also cause carboplatin resistance. Additionally, alterations in cellular pathways that are not directly related to platinum’s biochemical activity, such as EMTs, TME, and epigenetic changes, can cause carboplatin resistance. Figure created with BioRender.com. ALDH—aldehyde dehydrogenase; ATP7A/B—ATPase copper-transporting alpha/beta; Bcl2/X_L_—B-cell lymphoma 2/extra-large; BER—base excision repair; BRCA1/2—breast cancer 1/2; CAFs—cancer-associated fibroblasts; CTR1/2—cooper transporter 1/2; CXCR4—chemokine receptor 4; DNA—desoxyribonucleic acid; EMT—epithelial–mesenchymal transition; GSH—glutathione; HDAC1/10—histone deacetylase 1/10; HR—homologous repair; LAMA3—laminin alpha 3 expression; miRNAs—microribonucleic acid; ML/SH1/2—MUTL protein homolog 1/2; MMR—mismatch repair; MRP2/4—multidrug resistance-associated protein 2/4; MT—metallothionine; NCALD—DNA hypermethylation of neurocalcin delta; NER—nucleotide excision repair; NHEJ—non-homologous end joining; OC—ovarian cancer; OCT4—octamer transcription factor 4; SIRT5—sirtuin 5; SOX9—SRY-Box transcription factor 9; TAMs—tumor-associated macrophages; TILs—tumor-infiltrating lymphocytes; TME—tumor microenvironment; TWIST—twist-related protein 1; XIAP—X-linked inhibitor of apoptosis protein; ZIC1—zic family member 1.

Platinum resistance is a complex process that involves various factors that can hinder the effectiveness of platinum-based therapy. These factors can operate before or after platinum binds to its target. For example, reduced drug uptake or increased efflux can decrease the concentration of platinum inside the cell, known as pre-target resistance. Similarly, enhanced DNA repair mechanisms can overcome platinum–DNA binding, leading to on-target resistance. Moreover, mutations or changes in downstream signaling pathways that affect the cell’s ability to undergo apoptosis can cause post-target resistance. Additionally, alterations in cellular pathways that are not directly related to platinum’s biochemical activity, such as epithelial-mesenchymal transition (EMT) and epigenetic changes, can cause off-target resistance (Figure 1) [45].

#### 2.1.1. Pre-Target Resistance Mechanisms

##### Drug Influx and Efflux

Platinum agents cause cytotoxicity in cells when they accumulate inside them. One of the main reasons for resistance to these agents is the dysregulation of drug influx and efflux pumps/transporters, which reduces the amount of drugs that accumulate inside cells [46]. Platinum-based compounds can enter cancer cells by passive diffusion or CTR1-mediated import [47] and can be exported by ATPase copper-transporting alpha/beta (ATP7A/7B) and multidrug resistance-associated protein 2/4 (MRP2/4) [48,49].

CTR is a transmembrane influx transporter involved in copper homeostasis. It also plays an essential role in the intracellular uptake of platinum salts by facilitating the entry of drugs inside the cells. Consequently, its downregulation reduces treatment efficacy [50]. Studies have shown that deleting CTR1 can reduce the intracellular accumulation of cisplatin, leading to chemoresistance [51], while overexpression of CTR1 can increase sensitivity to platinum compounds [52,53]. It has been shown that cisplatin-sensitive A2780 cells express higher levels of CTR1 compared to cisplatin-resistant A2780CP cells [54]. Ishida et al. found that HGSC patients with platinum-sensitive disease at stage III or IV expressed significantly higher levels of CTR1 messenger ribonucleic acid (mRNA) than those with platinum-resistant or platinum-refractory disease [50]. On the other hand, cooper transporter 2(CTR2) is involved in regulating platinum levels inside the cells, acting as a platinum efflux transporter, and high levels of CTR2 are associated with platinum resistance in OC cells [55].

ATP7A sequesters platinum derivates to prevent their nucleus access, while ATP7B facilitates drug efflux [56,57]. Chisholm et al. showed that cells expressing high levels of ATP7A present low intracellular platinum concentration and exclude the drug from the nucleus [58]. ATP7A/B genes were expressed at higher levels in platinum-resistant cells compared with sensitive cells [57]. According to a study by Mangala et al. when ATP7B is silenced, it reduces cisplatin IC_50_ by 2.5 times and increases DNA adduct formation in cisplatin-resistant cells [57]. Patients with low ATP7B mRNA expression levels in colorectal cancer have better PFS and optimal curative effects from oxaliplatin plus 5-fluorouracil treatment compared to those with high ATP7B mRNA expression [59].

Many studies in OC have shown that high levels of MRP2 expression are linked with platinum resistance and adverse outcomes [49,60,61].

##### Detoxification Systems

Neoplastic cells have a higher level of intracellular reactive oxygen species (ROS) than normal cells, which plays a significant role in the pathogenesis [62]. Excess ROS production is caused by ionizing radiation and chemotherapeutic compounds, activating apoptosis pathways [63]. Cancer cells upregulate antioxidant enzymes like glutathione (GSH) and metallothionein (MT) to counteract the effect of ROS, which provides them with a survival benefit [64,65]. Several cancers, including OC, show high levels of GSH expression that is linked with increased activity of GSH-related enzymes and exporter proteins, such as γ-glutamyl-cysteine ligase, γ-glutamyl-transpeptidase, and GSH-transporting export pumps [66,67]. These enzymes have been associated with platinum resistance [66,67,68]. GSH has a high affinity toward platinum compounds, forming platinum-thiol conjugates, reducing platinum-induced oxidative stress levels, and decreasing the cytotoxicity of reactive platinum [69]. The ability of GSH transferase and MT to sequester and inactivate platinum compounds has been linked to platinum resistance by reducing their availability [64,70].

On the other hand, Criscuolo et al. found that cisplatin-resistant cells display lower levels of reduced GSH and reduced expression of enzymes involved in GSH biosynthesis and recycling. This is due to cisplatin binding, hindering GSH oxidation and accumulating ROS [71]. Interestingly, only platinum-sensitive cells showed a significant increase in ROS levels following cisplatin treatment, making them more susceptible to oxidative stress-induced cell death [71].

#### 2.1.2. On-Target Resistance Mechanisms

##### DNA Damage Repair

The first step towards the cytotoxicity of platinum-based agents is the formation of DNA adducts. Therefore, cell sensitivity is modulated by their capacity to recognize and repair drug-induced DNA damage [36,72]. This process is known as DNA damage response (DDR). It can activate one or more DNA repair pathways, including mismatch repair (MMR), base excision repair (BER), nucleotide excision repair (NER), homologous repair (HR), and non-homologous end joining (NHEJ) [72,73]. In many tumors, abnormal activity in one of these pathways is frequently observed, leading to increased capacity to repair or tolerate platinum-induced DNA damage, resulting in platinum resistance [36]. Moreover, the upregulation of DNA repair proteins may lead to removing platinum adducts and increased capacity to repair DNA damage [36]. Indeed, most platinum-resistant tumors display upregulation of DNA damage repair proteins, including MMR repair proteins (MSH1/2 and MLH1), excision repair cross-complementing (ERCC) proteins, and Fanconi anemia complementation group D2 (FANCD2), and also secondary mutations in BRCA1/2 genes [36,74,75,76,77].

The proteins encoded by BRCA1/2 are essential for DNA double-strand break (DSB) repair by the HR pathway [78]. Around 25% of OC patients have BRCA1/2 mutations, increasing their sensitivity to DNA-damaging agents, such as platinum and PARPis [79,80]. Some studies suggest that prolonged exposure to platinum may create selective pressure, leading to reversion mutations or intragenic deletions in BRCA1/2-mutated genes. This could restore the BRCA1/2 reading frame, produce a functional protein, and re-acquire HR proficiency as a possible mechanism of platinum resistance [77,81,82]. BRCA1/2 is also implicated in the response to replication stress. Pax transactivation domain-interacting protein (PTIP) is part of the histone methyltransferase complex protecting stressed replication forks. Loss of PTIP, PARP1, and chromodomain helicase DNA-binding protein 4 (CHD4) in a BRCA2-deficient background is associated with cisplatin resistance due to an increased protection of replication forks [83]. According to TheCancer Genome Atlas (TCGA) database, OC patients who had BRCA1/2 mutations and were treated with a platinum agent with a high level of PTIP had longer PFS, presumably reflecting the increased sensitivity of their cancer cells to chemotherapy [83]. Moreover, some studies suggest that cisplatin-resistant OC cells exhibit an enhanced NER pathway [84,85]. The expression levels of several NER proteins [e.g., xeroderma pigmentosum complementation group A/C/G (XPA/C/G), excision repair cross-complementation group 1–xeroderma pigmentosum complementation group F (ERCC1-XPF)] and somatic mutations in XPD significantly affect cellular sensitivity to cisplatin [86,87]. Indeed, the overexpression of XPC in several types of cancer cells (e.g., colorectal and gastric cancers) leads to elevated cisplatin resistance [86,87].

#### 2.1.3. Post-Target Resistance Mechanisms

##### Apoptosis and Cell Cycle Regulation

Most anticancer agents trigger cell death through processes such as apoptosis; however, cancer cells can develop mechanisms to avoid this process. Key apoptotic regulators and signaling pathways mediate cellular cytotoxic response to platinum agents, and consequently, deficiencies within the pathways contribute to chemoresistance [88]. Platinum compounds can trigger apoptosis by extrinsic and intrinsic pathways [89,90,91]. Proapoptotic signaling activation leads to mitochondrial outer-membrane permeabilization and release of cytochrome C that triggers the activation of the caspase cascade [92]. Platinum-resistant tumors can express high levels of anti-apoptotic proteins or have defects in mitochondrial signaling [35]. In OC, the B-cell lymphoma 2 (Bcl-2) family of apoptosis inhibitors, including Bcl-2 itself and B-cell lymphoma/extra-large (Bcl-X_L_), can be upregulated, preventing activation of apoptosis and allowing time for DNA repair [93,94,95,96].

X-linked inhibitors of apoptosis proteins (XIAPs) are the most potent among human anti-apoptotic proteins (IAPs). They can block apoptosis pathways by selectively binding and inhibiting caspase-3/7/9, decreasing chemotherapy-induced apoptosis [97]. In OC, several studies showed that XIAPs and survivin are overexpressed and contribute to platinum resistance [98,99].

#### 2.1.4. Off-Target Resistance Mechanisms

##### Tumor Microenvironment and Cancer Stem Cells

OC cells can survive in malignant ascites, which act as a liquid TME where neoplastic cells disseminate throughout the abdominal cavity [15]. TME is a complex ecosystem comprising normal cells such as stromal cells, immune cells, endothelial cells, adipocytes, bone-marrow-derived cells, and lymphocytes, as well as ECM components that contribute to tumor cell growth, differentiation, and invasiveness [100].

Non-tumor cells in the TME may contribute to the platinum resistance of OC cells. Indeed, the ECM signaling is dysregulated through the activation of cancer-associated fibroblasts (CAFs) and tumor-associated macrophages (TAMs), which lead to excessive ECM remodeling, promoting tumor progression and activating multiple signaling pathways that cause platinum resistance [101]. Two studies have found that cancer cells can stimulate mesothelial cells by releasing transforming growth factor beta (TGFβ). This stimulation causes the mesothelial cells to secrete either osteopontin [102] or fibronectin [103], which reciprocally stimulate survival signaling in the cancer cells. Additionally, collagen types I, V, and XI, presumably originating from fibroblasts, have been shown to contribute to the platinum resistance of OC cells [104,105]. CAF-induced resistance can also result from the secretion of specific cytokines, proteins, or exosomal miRNAs to activate anti-apoptosis-related signaling pathways, such as phosphoinositide 3-kinase/protein kinase B (PI3K/Akt), annexin A3/c-Jun N-terminal kinase (ANXA3/JNK), and interleukin-11/interleukin 11R/signal transducer and activator of transcription 3 (IL-11/IL-11R/STAT3) [106,107,108,109]. IL8 derived from OC cells or CAFs induces OC stemness, platinum [110], and taxane resistance [111]. Similarly, C-X-C motif chemokine 12 (CXCL12) derived from CAFs can induce EMT and cisplatin resistance [112]. Moreover, CAFs can also release cysteine and GSH, limiting intracellular platinum concentration [113,114]. Another player contributing to drug resistance is the cancer-associated adipocytes (CAAs) responsible for arachidonic acid secretion. This chemoprotective lipid mediator acts directly on OC cells and inhibits cisplatin-induced apoptosis through Akt pathway activation [115].

Some studies have indicated that platinum resistance might be caused by the interaction between ECM and cell adhesion molecules, such as CD44, CD117, CD133, and β-integrins, providing a survival advantage to tumor cells when exposed to cytostatic drugs [116]. Additionally, tumor-infiltrating lymphocytes (TILs), including regulatory T cells and TAMs, can promote platinum resistance. TAMs can cause platinum resistance by inducing EMT and ECM remodeling [101,117]. It has been observed that stromal cells can influence the sensitivity of cancer cells to chemotherapy, and spatial transcriptomics has shown the uneven intratumoral distribution of CAFs [118]. Therefore, the heterogeneous response of tumor cells to chemotherapy results from cancer cell-intrinsic factors and the spatial heterogeneity of stromal cells, which directly affect patient survival [118].

Overexpression of cancer stem cell (CSC) biomarkers is also associated with enhanced EMT features and chemoresistance [119]. These CSC markers include aldehyde dehydrogenase (ALDH), CD44, CD24, CD133, CD117, and chemokine receptor 4 (CXCR4) and are more prevalent in platinum-resistant OC cells compared to platinum-sensitive ones [120,121]. Additionally, in OC, an enhanced expression of stemness-maintaining proteins, including Nanog, octamer transcription factor 4 (OCT4), and CD73, are reported to be expressed in platinum-resistant cells [122,123,124].

##### Epigenetic Modifications

Epigenetic modifications are associated with acquiring a platinum-resistant phenotype through multiple mechanisms. Studies have extensively considered the role of DNA methylation in OC chemoresistance, and it has been observed that platinum-resistant samples have a higher frequency of hypermethylation as compared to platinum-sensitive [125,126]. Cardenas et al. have shown that EMT may be driven by aberrant methylation, resulting in the development of a platinum-resistant phenotype [125]. DNA hypermethylation of neurocalcin delta (NCALD) and reduced laminin alpha 3 (LAMA3) expression is also associated with EMT in HGSC, which is correlated with chemoresistance and poor patient outcomes [127]. Furthermore, hypermethylation of genes such as SRY-Box transcription factor 9 (SOX9), zic family member 1 (ZIC1), and twist-related protein 1 (TWIST), which are involved in EMT, is also associated with carboplatin-resistant OC [128]. The genes frizzled class receptor 1/10 (FZD1/10), and glycogen synthase kinase 3 beta (GSK3B) involved in the wingless/integrated (Wnt) signaling pathway have been found to have different levels of methylation in both platinum-resistant and platinum-sensitive samples [129]. Another study demonstrated that most differentially methylated sites were hypomethylated in the cisplatin-resistant cell lines compared to the sensitive ones [130]. Indeed, Bonito et al. showed that msh homeobox 1 (MSX1) transcription factor can influence EMT in OC and that DNA hypomethylation leads to decreased MSX1 expression, which is associated with cisplatin resistance. At the same time, MSX1 overexpression sensitizes cells to cisplatin [131].

Epigenetic modifications that contribute to resistance are not limited to DNA methylation. The overexpression of some histone deacetylases, including histone deacetylase 1/10 (HDAC1/10), and sirtuin 5 (SIRT5), has also been reported in OC platinum resistance [132,133,134,135]. Moreover, dysregulation of microRNAs (miRNAs) miR-214, miR-137, miR-21-3p, miR-199a, and miR-98-5p has also been linked to platinum resistance in OC [136,137,138,139,140]. Xiao et al. showed an inverse correlation between miR-139 and ATP7A/B expression, demonstrating that high expression levels of miR-139 sensitize OC cells to cisplatin-based chemotherapy through ATP7A/B regulation [141]. Moreover, Dwivedi et al. also showed that miR-15a and miR-16 restored cisplatin sensitivity by inhibiting ATP7B expression [142]. Similarly, the upregulation of miR-490-3p and downregulation of miR-411 were associated with increased cisplatin sensitivity via the inhibition of MRP2 [143,144]. MiR-514 also increases cisplatin chemosensitivity by targeting ABC family members ABCA1/10 and ABCF2 [145]. Furthermore, miRNAs can influence pathways involved in DNA repair, inducing platinum resistance. Indeed, miR-211 facilitated platinum-induced DNA damage by targeting DDR effector genes [i.e., DNA polymerase eta (POLH), tyrosyl-DNA phosphodiesterase 1 (TDP1), alpha-thalassemia retardation x-linked (ATRX), mitochondrial ribosomal protein S11 (MRPS11), and germline ERCC excision repair 6 like 2 (ERCC6L2)], enhancing platinum sensitivity in OC cells [146]. MiR-30a-3p and miR-770-5p target ERCC1/2, essential effectors of the NER pathway [147,148], and its upregulation restores cisplatin sensitivity [147,148]. Finally, miR-152 can increase platinum sensitivity by targeting RAD51 and suppressing HR [140].

### 2.2. Mechanisms of Paclitaxel Resistance in Ovarian Cancer

Paclitaxel belongs to the taxane family of compounds and is the first example of a microtubule-stabilizing class of antimitotic drugs successfully used to treat multiple neoplasms [149,150]. Microtubules are a dynamic network of α and β tubulin that play a critical role in the formation of mitotic spindle fiber, which is essential for chromosomal separation during M-phase [151]. Additionally, they are necessary for maintaining cell structure, motility, and cytoplasmic trafficking [152].

Paclitaxel suppresses microtubule dynamics by binding to the β-subunit of the tubulin heterodimer [153,154], which disrupts the assembly of mitotic spindles, induces mitotic arrest and aberrant mitosis, and subsequently leads to cell death [155,156]. It can also exert cytotoxic activity against cancer cells through non-mitotic mechanisms [157,158], including inducing phosphorylation of apoptotic protein Bcl-2 [159], disruption of microtubule-mediated intracellular transport [160] or physical breaking of the nuclear envelope by forming rigid microtubule bundles [161]. Moreover, paclitaxel can stimulate an inflammatory response by inducing nuclear fragmentation [162], and it possesses antiangiogenic activity by increasing the dynamic instability of interphase microtubules in endothelial cells and inhibiting the migration of these cells [163].

Paclitaxel is commonly used alone or in combination with carboplatin to manage OC and is considered the primary treatment option. However, the disease often returns and becomes resistant to this treatment [164,165]. Furthermore, the treatment may cause severe side effects, such as peripheral neuropathy, which may require dose de-escalation or treatment cessation [166,167]. To improve the effectiveness of treatment and potentially reverse resistance to paclitaxel, finding drug partners that can work together with paclitaxel is recommended.

**Figure 2:**
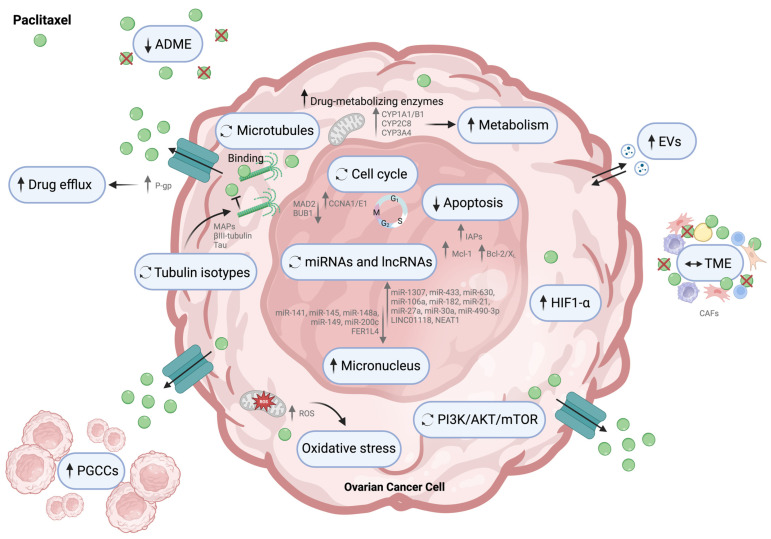
Schematic representation of the main molecular mechanisms contributing to paclitaxel resistance in OC. Paclitaxel resistance mechanisms in OC involve a decreased cellular drug accumulation by decreased ADME, increased drug efflux pumps (such as P-gp), and metabolizing enzymes (such as upregulation of CYP enzymes). Moreover, paclitaxel resistance can also be associated with microtubule structure, stability, and expression, including mutations in *β*-tubulin or altered isotype expression (such as increased MAPs). Changes in microtubule-binding proteins may underlie reduced drug-binding affinity. In addition, hypoxic conditions, the alteration of the drug target, deranged nuclear–cytoplasmic shuttling, and increased ability to counter drug-induced damage or apoptosis also contribute to taxane resistance. Cell cycle regulation by enhanced Bcl-2/X_L_, Mcl-1, and IAPs, oxidative stress by enriched ROS, TME, EVs, and multi-micronucleus also impact paclitaxel resistance. Modifications in the nuclear envelope, such as the reduction in/loss of lamin A/C proteins, were also found to be associated with paclitaxel-induced nuclear envelope breakage and the formation of micronuclei. Figure created with BioRender.com. ADME—absorption, distribution, metabolism, and elimination; Bcl2/X_L_—B-cell lymphoma 2/extra-large; BUB1—mitotic checkpoint serine/threonine kinase; CCNA1/E1—cyclin A1/E1; CYPs—cytochrome P450; EVs—extracellular vesicles; FER1L4—Fer-1-like protein 4; HIF1-α—hypoxia-induced factor 1-α; IAP—inhibitor of apoptosis protein; lncRNAs—long non-coding ribonucleic acid; MAD2—mitotic arrest deficiency protein 2; MAPs—microtubule-associated proteins; Mcl-1—myeloid leukemia 1; miRNAs—microribonucleic acid; OC—ovarian cancer; PGCCs—polyploid giant cancer cells; P-gp—P-glycoprotein; PI3K/AKT/mTOR—phosphoinositol three kinases/protein kinase B/mammalian target of rapamycin; ROS—reactive oxygen species; TME—tumor microenvironment.

In OC, paclitaxel-resistance mechanisms involve a decreased intracellular drug concentration (mediated by an increased drug export) [168], an increased metabolism of taxanes [a consequence of upregulating cytochrome (CYP) enzymes] and changes associated with microtubules’ structure, stability, and expression, including mutations in *β*-tubulin or altered isotype expression. Changes in microtubule-binding proteins may underlie reduced drug-binding affinity [169]. In addition, hypoxic conditions [169], the alteration of the drug target, deranged nuclear–cytoplasmic shuttling, and increased ability to counter drug-induced damage or apoptosis also contribute to taxane resistance [152]. Modifications in the nuclear envelope, such as the reduction in/loss of lamin A/C proteins, were also found to be associated with paclitaxel-induced nuclear envelope breakage and formation of micronuclei [170] that ultimately impact paclitaxel resistance (Figure 2).

#### 2.2.1. Intracellular Drug Concentration

Maintaining adequate intracellular levels of paclitaxel is crucial for exerting its cytotoxic effects. Still, the concentration of taxane in the cellular compartment can be influenced by various mechanisms that ultimately affect tumor cytotoxicity [171].

##### Solubility Absorption, Distribution, Metabolism, and Elimination

Different types of taxanes have varying solubility, absorption, distribution, metabolism, and elimination (ADME) properties. These differences result in distinct efficacy and drug effects that affect cell death [172]. Moreover, taxanes are hydrophobic compounds with low solubility, preventing them from being orally administered [173]. To overcome this limitation and optimize their effectiveness, formulations are being developed to improve their absorption and cellular uptake, reduce paclitaxel resistance, and minimize collateral toxicity [173].

##### Drug Efflux

P-gp is a drug efflux pump whose overexpression increases the efflux of its substrates, reducing their intracellular concentration and making them less effective [174]. These substrates include paclitaxel, and undeniably, one of the most well-known causes of taxane resistance is P-gp overexpression [175,176,177]. It has been negatively correlated with paclitaxel response, including in OC [176,177]. Studies have shown that chemoresistant OC tumors have higher P-gp levels than their chemosensitive counterparts [152,178].

To reverse paclitaxel resistance in OC, P-gp inhibitors such as verapamil and elacridar are being tested preclinically [179,180]. Unfortunately, most inhibitors are unsuitable because of the difficulty of explicitly inhibiting the P-gp binding domain, the toxicity related to the high doses required, and compensation by other drug pumps [179,180]. There is also a risk that P-gp inhibitors may inhibit some CYP enzymes essential to detoxify normal cells during chemotherapeutic treatment [18].

##### Metabolizing Enzymes

An alternative way taxane resistance can be developed is through increased drug metabolism. Paclitaxel is a substrate of the hepatic P450 enzymes CYP1A1/B1, CYP2C8, and CYP3A4 [181,182,183]. CYP enzymes are present in both healthy and cancer tissues. However, increased expression and oxidizing activity in neoplastic cells led to an increase in the hydroxylation of metabolites of taxanes [31,182]. As a result, the metabolized drugs have low levels of cytotoxic antitumor activity [31]. However, inhibiting CYP enzymes can cause other problems, such as other drugs not being properly metabolized, which can lead to unexpected or potentially dangerous drug–drug interactions [184].

CYP2C8 is the primary enzyme responsible for paclitaxel metabolism, and it is overexpressed in OC [182]. This enzyme could be an essential biomarker to predict paclitaxel response [31]. In addition, CYP3A is associated with taxane metabolism [185], and its inhibition by taxanes could lead to increased drug toxicity from other drugs, which are also CYP3A substrates [31].

A study using OC cell lines revealed an essential increase in CYP1A1 mRNA and protein expression compared to primary cultures or immortalized HOSE cell lines [186]. Similarly, the authors also found that various types of OC patients’ specimens showed a moderate-to-high cytoplasmic expression of CYP1A1 compared with benign epithelia [186]. McFadyen et al. found that most OC specimens investigated (92%) had significantly higher levels of CYP1B1 expression. They demonstrated a strong correlation between CYP1B1 expression in primary and metastatic OC [183]. Downie et al. also revealed that CYP1B1 was the only enzyme not detected in normal ovarian tissue, a finding consistent with previous studies showing the presence of CYP1B1 in OC cells without expression in normal tissue [187]. Another study showed that paclitaxel treatment induces CYP1B1 expression, and inhibiting CYP1B1 can reverse resistance to paclitaxel [188]. Preclinical studies have demonstrated that resveratrol and its analog (DMU-212) inhibit CYP1A1/B1 transcription, protein expression, and enzymatic activity [189,190,191]. However, further studies are needed to determine whether its biological effects are associated with reversing paclitaxel resistance.

#### 2.2.2. Microtubule Regulatory Proteins and Tubulin Isotypes

Considering that paclitaxel exerts its cytotoxic activity through binding to β-tubulin, a reduction in intracellular tubulin concentrations, point mutations in the gene(s) encoding tubulin, selective alterations in the expression of tubulin isotypes, and changes in microtubule-associated proteins (MAPs) are significant mechanisms of paclitaxel resistance [192,193].

The expression of different tubulin isoforms with other binding domains, for example, the upregulation of β3-tubulin, can influence drug resistance by altering the binding affinity of taxanes [31,194]. Usually, β3-tubulin is found only in neuronal tissues, but in epithelial tumor cells, it can be aberrantly expressed [195]. In many neoplasms, including OC, high levels of β3-tubulin have been associated with poor PFS, higher histological grade, and increased taxane resistance [166,193,196]. Therefore, high levels of β3-tubulin can inhibit the microtubule-stabilizing activity [197,198]. Conversely, when β3-tubulin is depleted, the sensitivity to paclitaxel can be increased [197,198].

Mutations in *β*-tubulin can reduce the affinity of paclitaxel [166,199]; however, studies evaluating patient samples have not found frequent mutations, suggesting that this molecular alteration is not a general mechanism for paclitaxel resistance [200]. The post-translational modification of tubulin, such as phosphorylation, acetylation, and detyrosination, can affect the organization and dynamics of microtubules. However, this has also not been found to occur frequently in clinical samples [166].

MAP4 and Tau proteins can competitively bind to microtubules at the same site as paclitaxel, making them potential modulators of resistance [201]. Smoter et al. evaluated clinical data from patients treated with platinum plus taxane and found a correlation between Tau expression and paclitaxel resistance. Tau may limit the access of paclitaxel to microtubules [201]. On the other hand, MAP4 facilitates microtubule assembly and stabilization by increasing the longitudinal interaction within filaments, preventing depolymerization of microtubules [202]. Therefore, alterations in the expression of MAP4 isoforms can affect the sensitivity of cancer cells to microtubule-targeting agents [202]. In the OC context, Poruchynsky et al. found a positive correlation between MAP4 phosphorylation and paclitaxel resistance [203]. Similarly, Yu et al. found that MAP kinase spleen tyrosine kinase (SYK) also mediated paclitaxel resistance [204]. The increase in taxane resistance results from the upregulation of SYK, leading to the phosphorylation of tubulin and MAP proteins [204]. Some studies indicate that SYK inhibitors offer an excellent alternative to target microtubules by preventing this phosphorylation and restoring paclitaxel sensitivity [31,205]. Stathmin, a microtubule-sequestering protein, induces microtubule destabilization. Its expression is markedly increased in taxane-resistant breast and ovarian tumors and was associated with an increased paclitaxel resistance and an unfavorable prognosis [206,207].

#### 2.2.3. Apoptosis and Cell Cycle Regulation

Alterations in specific protein expression within intrinsic and extrinsic apoptotic pathways can suppress apoptosis, which leads to increased DNA repair and high tolerance for genetic damage [208]. The intrinsic apoptosis pathway is the most prominent cell death signaling cascade, and the Bcl-2 protein family mainly controls it. This can be divided into pro-survival/anti-apoptotic (Bcl-2, Bcl-X_L_, Mcl-1, Bcl-W, BFL1), effectors (BAK, BAX, BOK), BH3-only activator (BIM, BID, PUMA), and sensitizer (NOXA, BAD, BMF, BIK, Hrk) proteins [209]. In OC, the overexpression of the pro-survival protein family members Bcl-X_L_, Bcl-2, and MCL-1 has been associated with worse prognosis [210] and resistance to taxane-induced apoptosis [93,211,212].

Survivin, a member of the IAP family, is often overexpressed in various cancers, including HGSC [213,214]. However, targeting survivin remains problematic due to its physiologic roles in mitosis, motility, and several other cellular pathways [215].

CCNE1 complexes with cyclin-dependent kinase 2 (CDK2) to regulate the transition from G_1_ to S phase, which marks the beginning of DNA replication and cell cycle initiation [216]. Increased CCNE1 leads to increased DNA synthesis and uncontrolled replication, enhancing the probability of chromosomal errors and genetic instability [31,217]. Moreover, cyclin A1 (CCNA1) expression increase is widely observed in paclitaxel-resistant OC patients, particularly those with HGSC [31,218,219]. Targeting CCNE1 is complex, and a more promising option proposes the indirect inhibition of CDK2 or PLK1 [31]. CDK2 inhibitors effectively suppressed pathway hyper-activation caused by CCNE1 [220]. Moreover, polo-like kinase 1 (PLK1; a key regulator in mitosis) inhibitors combined with paclitaxel showed an increased potential to induce apoptosis in HGSC cells with amplified CCNE1 [221].

Changes in spindle assembly checkpoint (SAC) proteins may also contribute to resistance to paclitaxel. To target taxane-resistant mitotic cells, it may be most effective to focus on the mechanisms responsible for mitotic slippage and mitotic catastrophe [31]. BUB1 mitotic checkpoint serine/threonine kinase (BUB1), BUBR1, and mitotic arrest deficiency protein 2 (MAD2) are proteins that play an impactful role in active SAC/mitotic arrest response [222]. Downregulation of these proteins [223] prevents paclitaxel-induced activation of the SAC and reduces subsequent apoptosis, thereby increasing resistance [224,225]. Mitosis exit is regulated by the relaxation of the SAC signal and degradation of cyclin B1 [222]. OC cells bypass the paclitaxel-induced mitotic arrest by weakening the SAC signal and effector proteins (31). In paclitaxel-resistant OC cells, decreased cyclin B and BUB1 expression disrupt SAC signal control during mitotic arrest [226]. Likewise, manipulating the anaphase-promoting complex/cyclosome (APC/c) and proteins that regulate mitotic exit may provide another therapeutic opportunity to overcome taxane-mediated cell arrest [31]. During paclitaxel-induced mitotic arrest, the SAC is activated, which results in the inhibition of APC/c. Consequentially, cyclin B1 is not degraded, sister chromatids do not separate, and microtubules attempt to reconnect with kinetochores [31,227]. However, paclitaxel treatment causes microtubule stasis, and cells remain in this “non-mitotic mitosis limbo” until they die or enter senescence [228]. To prevent cells from escaping mitotic arrest, some studies suggest that targeting polo-like kinase 1 (PLK1) may be a better alternative [31,229]. Noack et al. and Raab et al. showed that combining paclitaxel with an inhibitor for PLK1 can re-sensitize OC cells to the taxane agents [221,229]. Alternatively, inhibiting the SAC may allow paclitaxel-arrested cells to abort mitosis, resulting in polyploid cells. One way to indirectly achieve this is by stimulating branched-chain amino acid metabolism, leading to the inactivation of mammalian target of rapamycin complex 1 (mTORC1) and Aurora kinases [230].

#### 2.2.4. Signal Transduction Pathways

The phosphoinositol three kinases/protein kinase B/mammalian target of rapamycin (PI3K/AKT/mTOR) pathway is widely known to be altered in a great majority of cancers, including OC. It can contribute to taxane resistance [101,231] and suppresses apoptosis, but some studies have shown that specific path inhibitors (e.g., AZD8835, AZD8186, and D-11688) can sensitize resistant cells to taxanes [232,233].

#### 2.2.5. Oxidative Stress

ROS-dependent resistance to taxanes can also occur by redox-responsive transcription factors [e.g., nuclear-factor kappa beta (NF-κB), protooncogene jun (c-Jun), nuclear factor erythroid 2-related factor 2 (Nrf2), and hypoxia-inducible factor 1 subunit alpha (HIF-1α)], which activate the cellular antioxidant systems and increase the expression of survival proteins [234]. Other ROS-dependent mechanisms, such as a switch from apoptosis to autophagy, EMT stimulation, and differentiation of CSCs by ROS, can also contribute to taxane resistance [235].

#### 2.2.6. Cellular Homeostasis and Glucose Metabolism

Tumor cells have increased metabolic demands because they require a continuous supply of glucose and amino acids to support cell proliferation [171]. The altered glucose metabolism is an essential source of metabolic plasticity that helps tumor cells undergo sustained growth and proliferation and acquire chemoresistance [236]. Hypoxia, a prominent feature of solid tumors, is not per se a driver mechanism of taxane resistance; however, it affects several cellular pathways, activates multiple resistance mechanisms, and mediates reduced apoptosis following taxane treatment [31,237]. Hypoxia-induced factor 1-α (HIF1-α) alters the expression of proteins, including cyclins, TGF-β, and c-Jun N-terminal kinase (JNK), that ultimately reduce taxane-induced G_2_-M arrest and apoptosis [31,237,238]. Moreover, tumor cells can inhibit apoptosis in adverse oxygen conditions by interacting with HIF1-α and members of the Bcl-2 family [239]. Under hypoxic conditions, HIF1 is stabilized, translocates to the nucleus, and activates target gene expression that promotes resistance to anticancer therapy by regulating metabolism, survival, drug efflux, signaling, and DNA repair [238]. Indeed, HIF1-α upregulation induces the expression of proteins associated with stemness, which increases resistance to taxane-induced apoptosis [238]. It will be worthwhile to consider targeting pathways downstream of HIF1, which may not be involved in normal cell metabolism but are involved in cancer cell metabolism [31].

#### 2.2.7. MicroRNAs and LncRNAs

Several studies in OC samples have shown that overexpression of miR-1307, miR-433, miR-630, miR-106a, miR-182, miR-21, miR-27a, miR-30a, and miR-490-3p, as well as the downregulation of miR-141, miR-145, miR-148a, miR-149, and miR-200c are associated with the development of taxane resistance [171,240,241,242]. On the other hand, the upregulation of miR-29b, miR-199a, miR-200a, miR-200c, and miR-215, along with the downregulation of miRNA-146a and miR-194 are associated with increased sensitivity to taxanes [171,242,243,244].

Regarding long non-coding ribonucleic acids (lncRNAs), the downregulation of Fer-1-like protein 4 (FER1L4) and the overexpression of long intergenic non-protein coding RNA 1118 (LINC01118) and nuclear enriched abundant transcript 1 (NEAT1) lead to paclitaxel resistance [245,246,247]. Although many other studies have documented the relevance of miRNAs and lncRNAs in developing taxane resistance in OC, targeting more than one miRNA or lncRNA might be necessary to achieve a therapeutic response. Nevertheless, these miRNAs and lncRNAs can serve as biomarkers to predict taxane response [31].

#### 2.2.8. Tumor Microenvironment

The TME can also contribute to chemoresistance to multiple antineoplastic drugs, including paclitaxel [248]. These effects are achieved by diverse signaling interactions, possibly via direct cell-to-cell contact or the release of soluble factors that promote cancer dissemination and multidrug resistance [249]. Zhang et al. demonstrated that adipose stromal cells derived from the omental tissue of patients with OC promoted paclitaxel and carboplatin resistance when co-cultured with various OC cell lines [250]. CAFs, one of the most critical immunosuppressive cells within TME, can secrete IL-6 and promote TGF-β-mediated EMT in OC via the Janus kinase/signal transducer (JAK2/STAT3) pathway. This leads to the inhibition of apoptosis and subsequent paclitaxel resistance [251]. Leung et al. showed that the CAF-derived factor, microfibrillar-associated protein 5 (MFAP5), increases the expression of lipoma-preferred partner in microvascular endothelial cells, leading to alterations in endothelial cell permeability and compromised paclitaxel delivery to tumor cells [252].

#### 2.2.9. Extracellular Vesicle-Dependent Intercellular Communications

Extracellular vesicles (EVs) are small structures secreted by cells and can contain bioactive molecules, proteins, RNA, and DNA. Other cells can take them up and play an essential role in intercellular signaling [253]. Cancer cells can secrete high amounts of EVs, and some studies showed that their molecular content mediates resistance to many chemotherapeutic drugs [254,255]. Indeed, docetaxel resistance can be transferred via the horizontal transfer of EVs containing miRNAs, including miR-9-5p, miR-195-5p, and miR-203a-3p [256,257]. Lv et al. showed in breast cancer that EVs can sequester chemotherapeutic drugs, transport proteins, such as P-gp, and transfer taxane chemoresistance from cell to cell [258].

#### 2.2.10. Multi-Micronucleus and Laminin A/C Expression

Recent evidence suggests that paclitaxel can also operate as an anticancer therapy through a non-mitotic that forms multi-micronucleated cells [161], impacting interphase cells [259]. Micronuclei result from the physical breaking of nuclei and are unstable and readily ruptured to release enclosed DNA [260]. Paclitaxel-induced aberrant mitosis generates multiple nuclear lobules and micronuclei that trigger apoptosis [161]. Indeed, cancer cells often have a defective and flexible nuclear envelope that presents a deformed nuclear morphology and the tendency to undergo transient rupture [261]. Lamin A/C proteins are crucial in maintaining a sturdy nuclear envelope structure [262] and are usually lost, reduced, or heterogeneously expressed in OC [263]. Recently, Smith et al. showed that paclitaxel caused multi-micronucleation in malignant OC cells but not in normal cells, as well as susceptibility to undergo nuclear fragmentation and cell death correlated with a reduction in the nuclear lamina protein lamin A/C [264]. In lamin A/C-deleted mice, cells lose physical strength, become easily deformed, are susceptible to paclitaxel, and undergo nuclear breakage [264]. On the other hand, lamin A/C overexpression reinforces nuclear envelope structure and increases the resistance to paclitaxel-induced nuclear breakage in cancer cells [264]. Indeed, paclitaxel induces nuclear breakage in cancer cells with a malleable nucleus but not in normal cells with a stiffer nuclear envelope [264].

#### 2.2.11. Polyploid Giant Cancer Cells

Polyploid giant cancer cells (PGCCs) are observed in HGSC samples after chemotherapy and are associated with paclitaxel resistance [265,266,267]. Previously, they were thought to be nonviable cells due to their senescent nature and inability to execute mitosis [268]. However, PGCCs are viable cells and can grow progressively by amitotic budding, splitting, and bursts of proliferation from mononucleated or multinucleated giant cells [265]. To further study the role of PGCCs in OC cells treated with paclitaxel, Niu et al. analyzed the mRNA expression profile of PGCCs. They found that IL-6 activation dominated the senescence-associated phenotype in these cells. These researchers confirmed the relevance of these findings by blocking IL-6 and showing that it prevented PGCC formation and inhibited tumor growth in a patient-derived xenograft HGSC model [269].

### 2.3. Mechanisms of Resistance to PARP Inhibitors in Ovarian Cancer

PARP is an enzyme partly responsible for the correct repair of DNA damage. However, in cells with defective HR repair systems, DSBs are repaired by NHEJ, which is an error-prone process that can result in cell death [270].

PARPi has two main effects. First, it inhibits PARP1’s catalytic activity, which prevents the formation of PAR chains (PARylation) that recruit more DNA repair proteins. Second, it traps PARP1 by stopping its release from damaged DNA, thus halting the progression of replication forks [271].

Around 50% of HGSC tumors have defective HR. PARPis are now available to patients in the first-line and recurrent platinum-sensitive disease [272]. HR-deficient phenotype can result from germline or somatic mutation in BRCA1/2 (20% of the HGSC patients), non-mutational changes (such as BRCA1 promoter methylation), and mutations in other repair-associated genes [e.g., BRCA1 interacting helicase 1 (BRIP1) and RAD51C, which collectively account for 2% of the HGSC cases] [273,274]. The use of PARPis has significantly improved outcomes in many HGSC patients in recent years. However, the benefits are most evident in patients with germline or somatic BRCA1/2 mutations and patients with non-BRCA-related HR deficiency [275]. Despite the remarkable clinical benefit of PARPis in patient OS [276], acquired PARPi resistance has emerged as a critical challenge in improving treatment success in most patients with advanced HGSC [277,278]. In platinum-sensitive recurrent HGSC, the response rate to PARPi monotherapy was about 30–45% in BRCA1/2 mutation patients [279]. Patients with HR-proficient tumors present minimal benefits to PARPis as they present an innate resistance to them [277].

**Figure 3:**
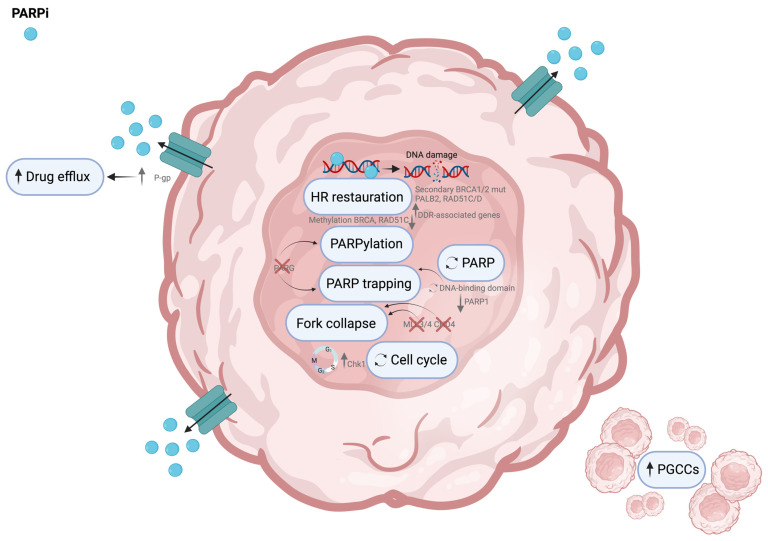
Schematic representation of the main molecular mechanisms contributing to PARPi resistance in OC. OC cells can develop PARPi resistance through various mechanisms, including the restoration of HR repair activity (alterations in BRCA1 and MMR genes) and replication stress mitigation, whereby the cancer cell slows the cell cycle and stabilizes replication forks. Moreover, mutations in PARP itself, genomic events that change protein poly-ADP-ribosylation (PARylation), PARP trapping, upregulation of drug efflux pumps (P-gp overexpression), and activation of alternate pathways can also alter PARPi response, leading to PARPis resistance. Figure created with BioRender.com. BRCA1/2—breast cancer 1/2; CHD4—chromodomain helicase DNA-binding protein4; Chk1—cell cycle checkpoint kinase 1; DDR—deoxyribonucleic acid damage response; DNA—deoxyribonucleic acid; HR—homologous recombination; MMR—mismatch repair; OC—ovarian cancer; PALB2—partner and localizer of BRCA2; PARG—poly(ADP-ribosyl) glycohydrolase; PARP1—polyadenosine diphosphate ribose polymerase 1; PARPis—polyadenosine diphosphate ribose polymerase inhibitors; PGCCs—polyploid giant cancer cells; P-gp—P-glycoprotein; RAD51C/D—RAD51 recombinase C/D.

PARPi resistance mechanisms in OC can be categorized into two main groups: those associated with HR and those not. Additionally, OC cells can develop PARPi resistance through various mechanisms, including the restoration of HR repair activity; replication stress mitigation, whereby the cancer cell slows the cell cycle and stabilizes replication forks; and mechanisms not currently assigned to a single DNA repair pathway-related process but still alter the response to PARPi, such as mutations in PARP itself, genomic events that change protein poly-ADP-ribosylation (PARylation), PARP trapping, upregulation of drug efflux pumps and activation of alternate pathways (Figure 3) [280].

#### 2.3.1. Resistance Mechanisms Associated with Restoration of Homologous Recombination

##### Homologous Recombination

Platinum-resistant tumors have a higher capacity for DNA repair. Patients previously exposed to platinum agents may also show PARPi cross-resistance [281]. Platinum sensitivity, as defined by PFI, is one of the best response indicators to PARPis. However, even among BRCA-mutated tumors with a PFI longer than six months, around 30% of patients may still show intrinsic resistance to PARPis [282,283]. On the other hand, for platinum-resistant BRCA-mutated tumors, the overall response rate to PARPis is around 30–40% [282]. PARPi undermines SSB damage repair, either by trapping PARP proteins on the DNA site of the lesion or by blocking PARP catalytic domain [284]. Unrepaired SSBs lead to DSBs and genomic instability, which triggers cell death [278]. Synthetic lethality due to the accumulation of DSB occurs in cells exposed to PARPis and has impaired HR repair machinery [285,286,287]. Restoring HR in HR-deficient tumors represents the most common acquired resistance mechanism to PARPis [21,288]. The restoration of HR activity can be achieved by directly affecting the HR machinery through genomic, epigenetic, and post-translational alterations or indirectly by growth factor receptor-mediated signaling pathways that increase the expression or activity of HR machinery [289]. The direct restoration of HR includes secondary mutations such as germline or somatic insertion or deletion mutations in BRCA1/2 that restore the open reading frame of the BRCA gene, remove the original deleterious mutation, and restore the expression of a functional protein [290,291]. Indeed, reversion mutations are one of the most prevalent and well-known causes of PARPi resistance [292,293]. Somatic reversions have also been observed in other HR pathway genes, such as partner and localizer of BRCA2 (PALB2), RAD51C, and RAD51D, and are associated with poor prognosis [290,294].

Epigenetic regulation involving the reduced promoter methylation of BRCA1 and RAD51C restores their functional expression and leads to PARPi resistance in HGSC [295]. Oncogenic signaling pathways, including the vascular endothelial growth factor receptor (VEGFR), PI3 kinase, and heat-shock protein 90 (HSP90), can also promote HR proficiency indirectly by increasing the expression of DDR-associated genes [289].

##### PARP Functions

PARP inhibition impairs DNA replication by generating PARP-DNA adducts; however, downregulation of PARP1 or alterations in its DNA-binding domains renders inhibitors of the PARP enzyme ineffective for inducing PARP trapping [296]. Furthermore, PARP1 binds to the damaged DNA through its zinc finger DNA-binding domain that can be modified by the allosteric effects of PARPis joining at the catalytic site [297]. Moreover, mutations or post-translational modifications in PARP1 were linked to a diminished PARP1 trapping activity on DNA and have also been uncovered as a mechanism of resistance [296,297].

The poly(ADP-ribosyl) glycohydrolase (PARG) enzyme also regulates PARP1 trapping by counterbalancing the activity of PARP1. PARG catabolizes PAR chains, the product of PARP activity [298]. PARylation is crucial for DNA repair by the HR and is reversed by PARG. Therefore, the loss of PARG can restore PARylation and PARP trapping and consequentially cause PARPi resistance [298].

#### 2.3.2. PARPi Resistance Mechanisms Not Associated with Homologous Recombination

##### Replication Fork

Replication fork degradation is an essential contributor to the synthetic lethality mechanism underlying the activity of PARPis; in contrast, the stabilization of stalled replication forks confers PARPi resistance [299]. The protection of stalled replication forks is a function usually performed by BRCA1/2 and PARP1. When BRCA1/2 are lost, the loss of replication fork protection allows cells to proceed unchecked into G2 and mitosis when, ultimately, replication forks collapse and cause cell death [277]. Therefore, when PARPi traps PARP on SSBs and replication forks stall, cells must rely on BRCA1/2 for survival [277]. Studies in BRCA-deficient cell lines showed loss of the mixed-lineage leukemia protein 3/4 (MLL3/4) complex protein PTIP or nucleosome remodeling factor CHD4, which leads to fork collapse [83]. Both these proteins are involved in recruiting the meiotic recombination 11 (MRE11) nuclease to the site of a stalled replication fork; in the absence of MRE11, newly synthesized DNA single strands are protected from degradation; cells with stalled replication forks do not enter mitosis and become resistant to PARPi [83,300].

Schlafen 11 (SLFN11) is another important mediator of PARPi resistance. It acts at the G1/S checkpoint by binding to a stressed replication fork and eventually causes irreversible cell cycle arrest and death [301]. In cells with BRCA1/2 mutation, SLFN11 depletion allows the fork to progress through G1/S, diminishing the efficacy of PARPis [302].

##### Drug Efflux Pumps

Increased drug efflux is a well-described mechanism of PARPi resistance [288]. Mutations in overexpression of ABCB1 that result in increased transcription of the drug efflux pump P-gp were found in tissue samples from PARPi-treated breast cancer and OC [303]. In paclitaxel-resistant OC cells, P-gp expression and gene copy numbers increased [304]. Indeed, PARPis, such as olaparib and rucaparib, are P-gp substrates, and prior treatment with paclitaxel may induce P-gp upregulation and indirectly induce PARPi resistance [304]. These findings hold immense clinical significance, highlighting the need for careful analysis when prescribing specific PARPis to OC patients. Administering these drugs as a second-line or maintenance treatment may not be effective in some patients due to the paclitaxel-induced resistance and the consequent active efflux of both drugs from cells, compromising the clinical response. Therefore, evaluating P-gp expression in patients who have failed paclitaxel therapy before administering a PARPi that is also a P-gp substrate [179] would be prudent.

##### Cell Cycle Regulation

For proper execution of DNA repair, cell cycle regulation is critical [305]. Upon recognition of DNA damage, the checkpoint ataxia–telangiectasia-mutated (ATM) and Rad3-related (ATR) kinases control downstream signaling pathways to determine whether the cell cycle progresses or is interrupted to allow for DNA repair [306]. This genotoxic stress-induced cell cycle arrest is implemented by cell cycle checkpoint kinases 1/2 (Chk1/2), activated by short-term and chronic replication stress, respectively [277]. Considering that approximately 95% of HGSC patients harbor a p53 mutation [19], causing a dysfunctional G_1_/S checkpoint, cancer cells depend on Chk1-mediated G_2_/M cell cycle arrest for DNA repair [307]. ATR mediates the G2/M cell cycle arrest and allows HR to repair DSBs in the presence of a collapsed replication fork [277]. Chk1 also phosphorylates BRCA2 and RAD51 recombinase to DSBs to facilitate HR. Therefore, inhibition of Chk1 impairs G2 arrest, preventing DNA repair via HR and leading to apoptosis [308,309].

##### Polyploid Giant Cancer Cells

A newly described PARPi resistance mechanism relies on activating the giant cell life cycle, leading to whole genome reprogramming in response to catastrophic stress, known as PGCCs. Zhang X. et al. developed an elegant experimental work to understand the mechanism of acquired resistance associated with PARPis in HGSC-derived organoids and patient-derived xenografts [310]. They showed that PGCCs exhibit features of senescent cells but, after olaparib withdrawal, can escape senescence via the restitution of multipolar endomitosis and other noncanonical modes of cell division to generate mitotically competent resistant daughter cells [310]. The authors also found that mifepristone (antiprogestin contraceptive drug) synergistically acts with olaparib to promote apoptosis of cells that are undergoing endoreplication, resulting in the inability to form PGCCs. This mechanism suggests that mifepristone may be more effective in killing newly formed PGCCs induced by therapeutic stress than preexisting PGCCs in patient tumors [310]. These authors presented a proof concept on how targeting PGCCs may represent a promising approach to overcome PARPi-induced resistance. Importantly, the authors looked for an association between p53 and BRCA mutational status and its association with PGCCs associated with olaparib resistance. Interestingly, although mutation or loss of p53 can sensitize the cancer cell to polyploidization due to defective cell checkpoints, the authors demonstrated the formation of PGCCs in all cell lines regardless of p53 mutations [310]. Additionally, using patient-derived xenografts PDX models with acquired olaparib resistance from both BRCA1 mutation and BRCA WT HGSC patient tumors, they found that PGCCs were more common in the olaparib-treated PDXs, suggesting that PGCCs, not BRCA status, are associated with acquired resistance to olaparib in patients with relapsed OC [310]. These results are consistent with the previous work reporting an increased ploidy in PARPi-resistant OC samples [311].

### 2.4. Mechanisms of Resistance to Bevacizumab in Ovarian Cancer

Tumorigenesis is a proliferative process highly dependent on developing a new vascular supply [312]. Angiogenesis refers to the stimulation of the growth of endothelial cells, which give rise to more blood vessels. It is a crucial factor in the progression of solid tumors and metastases because it supplies nutrients and oxygen and removes metabolic wastes [312]. Particularly in OC, angiogenesis induces malignant ascites formation and the spread of metastases, which lead to poor prognosis [313]. Therefore, angiogenesis has been an essential focus for the targeted treatment of OC.

The VEGF and VEGFR pathway are crucial regulators of angiogenesis, including in OC [313]. VEGF is produced by cancer cells to drive blood vessel growth and deliver oxygen and nutrients directly to tumors [314]. VEGF signaling is highly activated and closely associated with widespread intraperitoneal carcinoma and the formation of large malignant ascites volumes in OC [313,315]. VEGF gene expression has been found in OC tissue and omental metastases, malignant ascites, and the sera of patients with OC [316].

Bevacizumab is a humanized anti-VEGF monoclonal antibody that inhibits angiogenesis. It was the first angiogenesis inhibitor used in oncology clinical practice and the first approved for OC patients [317]. The FDA has approved bevacizumab as a first-line combination therapy with carboplatin/paclitaxel and a second-line strategy in platinum-sensitive or platinum-resistant OC [318,319,320]. Bevacizumab prevents the interaction between circulating VEGF and VEGFR, destroying existing vessels, disturbing neovascularization, releasing intratumor pressure, and consequently inhibiting angiogenesis [314]. Although several clinical studies have shown improvements in PFS in a subset of patients with advanced OC [320], the improvement in OS was not obvious, the duration of activity was relatively short (only 3–8 months in monotherapy), and the individual discrepancies and widespread resistance greatly limited the effectiveness of antiangiogenic therapy [321,322].

**Figure 4:**
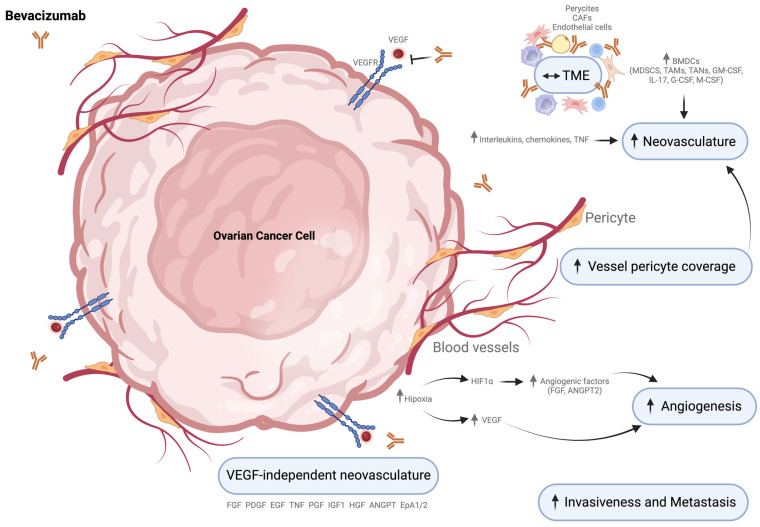
Schematic representation of the main molecular mechanisms contributing to bevacizumab resistance in OC. Bevacizumab resistance includes pharmacodynamic tolerance, tachyphylaxis, alteration of the neovascular architecture, redundant angiogenic factors, induction of hypoxia, increased tumor invasiveness, and metastatic behavior. Additionally, alterations in the ratio between VEGF-dependent and VEGF-independent vascular subtypes during antiangiogenic therapy led to bevacizumab resistance. Figure created with BioRender.com. ANGPTs—angiopoietins; CAFs—cancer-associated fibroblasts; EGF—epithelial growth factor; EpA1/2—ephrins; FGF—fibroblast growth factor; G-CSF—granulocyte colony-stimulating factor; GM-CSF–granulocyte-macrophage colony-stimulating factor; HGF—hepatocyte growth factor; HIF1-α—hypoxia-induced factor 1-α; IGF1—insulin-like growth factor 1; IL-17—interleukin 17; M-CSF—macrophage colony-stimulating; MDSCs—myeloid-derived suppressor cells; OC—ovarian cancer; PDGF—platelet-derived growth factor; PGF—placenta growth factor; TAMs—tumor-associated macrophages; TANs—tumor-associated neutrophils; TGF—transforming growth factor; TME—tumor microenvironment; TNF—tumor necrosis factor; VEGF—vascular endothelial growth factor; VEGFR—vascular endothelial growth factor receptor.

Bevacizumab-resistant mechanisms have been studied in different types of neoplasms and include pharmacodynamic tolerance, tachyphylaxis (i.e., acute decrease in response to a drug after a repetitive administration), alteration of the neovascular architecture, redundant angiogenic factors, induction of hypoxia, increased tumor invasiveness, and metastatic behavior [288,323,324]. Additionally, alterations in the ratio between VEGF-dependent and VEGF-independent vascular subtypes during antiangiogenic therapy led to resistance (Figure 4) [325].

#### 2.4.1. VEGF-Independent Revascularization

Resistance to bevacizumab can occur through the reactivation of tumor revascularization by “VEGF-independent pathways”. Antiangiogenic inhibitors can increase hypoxia, which then upregulates the production of other proangiogenic factors [326] such as fibroblast growth factor (FGF), platelet-derived growth factor (PDGF), epithelial growth factor (EGF), TGF, tumor necrosis factor (TNF), placenta growth factor (PGF), insulin-like growth factor 1 (IGF1), hepatocyte growth factor (HGF), angiopoietins (ANGPT), and ephrins (EpA1/2), among others [327]. PGF has been identified as a potential contributor to anti-VEGF resistance because its upregulation has been observed in patients receiving anti-VEGF [328]. Therefore, targeting only the VEGF pathway may not be enough to prevent angiogenesis, as other angiogenic factors or pathways can compensate to stimulate angiogenesis, ultimately leading to resistance.

#### 2.4.2. Neovasculature

HGSC patients exhibiting an immunoreactive profile trigger an immune response, which results in the recruitment of pro-angiogenic monocytes from the bone marrow, increased intratumoral hypoxia, upregulated HIF-1α, and a high pericyte coverage of the tumor vascular system. This results in an inefficient response to antiangiogenic drugs [329,330].

In solid tumors, angiogenesis leads to a defective vasculature with increased vascular and tumor permeability, consequently changing the TME and affecting intra-tumoral drug delivery [331]. Long-term antiangiogenic therapy significantly alters the expression of angiogenic factors, causing to an extensive morphological change in the vessels, and this remodeled neovascular architecture leads to treatment failure [327]. Recently, Arjaans et al. demonstrated in OC xenograft that bevacizumab decreased tumor uptake of antiangiogenic drugs while increasing the number of vessels pericytes [331,332]. Increased pericyte coverage in the tumor vasculature is a consequence of antiangiogenic treatment but is also a mysterious mechanism [333].

#### 2.4.3. Hypoxia

Antiangiogenic therapies result in vascular regression and can increase intra-tumoral hypoxia levels, leading to an abnormal upregulation of HIF1-α that can stimulate tumor and stromal cells to secrete large amounts of angiogenic factors, such as FGF and ANGPT2 [334]. This exacerbates vascular disorders and accelerates non-productive angiogenesis in an endless cycle of self-enhancement. As a result, therapy resistance increases, and the risk of disease progression becomes higher [334]. Additionally, antiangiogenic therapy can cause genetic alterations and hypoxia in the TME, which can lead to VEGF upregulation and the rebound of tumor angiogenesis. Other factors related to inflammation, immunosuppression, and bone marrow-derived cell (BMDC) recruitment, such as interleukins, chemokines, and TNF, have also been shown to be upregulated in patients treated with antiangiogenic therapy. These factors contribute to the evasion of antiangiogenic therapy by promoting neovascularization and enhancing aggressive malignant behaviors in tumor cells [333].

#### 2.4.4. Tumor Microenvironment

In the TME, apart from tumor cells, the increased infiltration of BMDCs, such as myeloid-derived suppressor cells (MDSCs), TAMs, and tumor-associated neutrophils (TANs), mediated by various attractants, such as GM-CSF, IL-17, G-CSF, and M-CSF, lead to neovascularization and immunosuppression induction, and allow tumor cells to escape antiangiogenic therapy [333]. Local stromal cells, including pericytes, CAFs, and endothelial cells, are also involved in tumor escape [333]. Endothelial cells are the central components of blood vessel walls and can be activated by various proangiogenic factors to initiate angiogenesis. Moreover, they can become refractory to antiangiogenic therapy by altering biological functions, cellular phenotypes, and secretory protein expression [333].

#### 2.4.5. Tumor Invasiveness and Metastasis

Antiangiogenic inhibitors block tumor growth. However, they also have been linked with increased local invasiveness and distant metastasis in different neoplasms [324,335,336]. For instance, in renal cell carcinoma and glioblastoma, tumor cells showed increased proliferation and became more invasive after being treated with bevacizumab [335,336].

## 3. Conclusions and Clinical Perspectives

The term “platinum-resistant OC” is used to refer to the recurrence of OC within six months of first-line platinum-based chemotherapy. Still, this definition is not entirely accurate as it does not oversee the tumor complexity and is based on outdated methods of detection [337]. Recent trials for platinum-resistant OC have not shown any clinically significant effect on PFS or OS since the approval of bevacizumab in combination with chemotherapy [338]. Nevertheless, these negative outcomes provide valuable insights into platinum-resistant OC and highlight the importance of discovering biomarkers in this clinical setting.

Drug resistance is a complex phenomenon that involves various mechanisms of resistance, some of which are not yet fully understood. This makes it difficult to develop effective therapeutic options to reverse chemoresistance and improve survival rates in OC patients. Cancer cells undergo a process of adaptation with each round of chemotherapy, which highlights the importance of evaluating the specific molecular profile of tumor cells during relapse or disease progression to treat each patient according to each resistance mechanism. However, access to tumor cells is limited, as patients rarely undergo a second surgery. It may be possible to obtain tumor samples through repeated biopsies, blood samples, or by analyzing malignant ascites cells and their supernatant components, such as proteins, metabolites, and extracellular vesicles, among others. By identifying the molecular portraits of chemoresistant tumors in these samples during disease progression, we could find new biomarkers capable of anticipating the clinical identification of resistant tumors and indicating a change in treatment towards more effective drugs. Patients with chemoresistant tumors often develop high volumes of malignant ascites, which can be used as a source of biological material to identify the type of resistance. This can be performed by evaluating the expression of drug efflux pumps (such as P-gp), detoxification enzymes (such as ALDH1), or detecting RAD51 status in cytologic samples. This approach can be easily performed in a pathology laboratory. Another approach could be the development of drug tests in malignant ascites cells to select the best treatment for each patient.

In this review, we performed an in-depth analysis of the resistance mechanisms commonly linked with the treatments used in OC. These include drug inactivation, alterations in drug targets, increased drug efflux pumps, a stronger capacity for DNA damage repair, and reduced drug absorption/accumulation. It is important to gain a comprehensive understanding of these molecular mechanisms that contribute to drug resistance in order to identify new biomarkers that can predict and monitor disease progression and discover new therapeutic targets.
